# Supplementary material for: Splice-Junction-Based Mapping of Alternative Isoforms in the Human Proteome
Source: Cell Rep. Author manuscript; Available in PMC 2020 Jan 15. (PMC6961840; doi:10.1016/j.celrep.2019.11.026)
Supplement: 2 [file NIHMS1546469-supplement-2.zip › DF1/PXD009737/TestisTrypsin/TestisTrypsin_25_PARK7_AGGPYDVVVLPGGNLGAQNLSESAAVK.pdf]

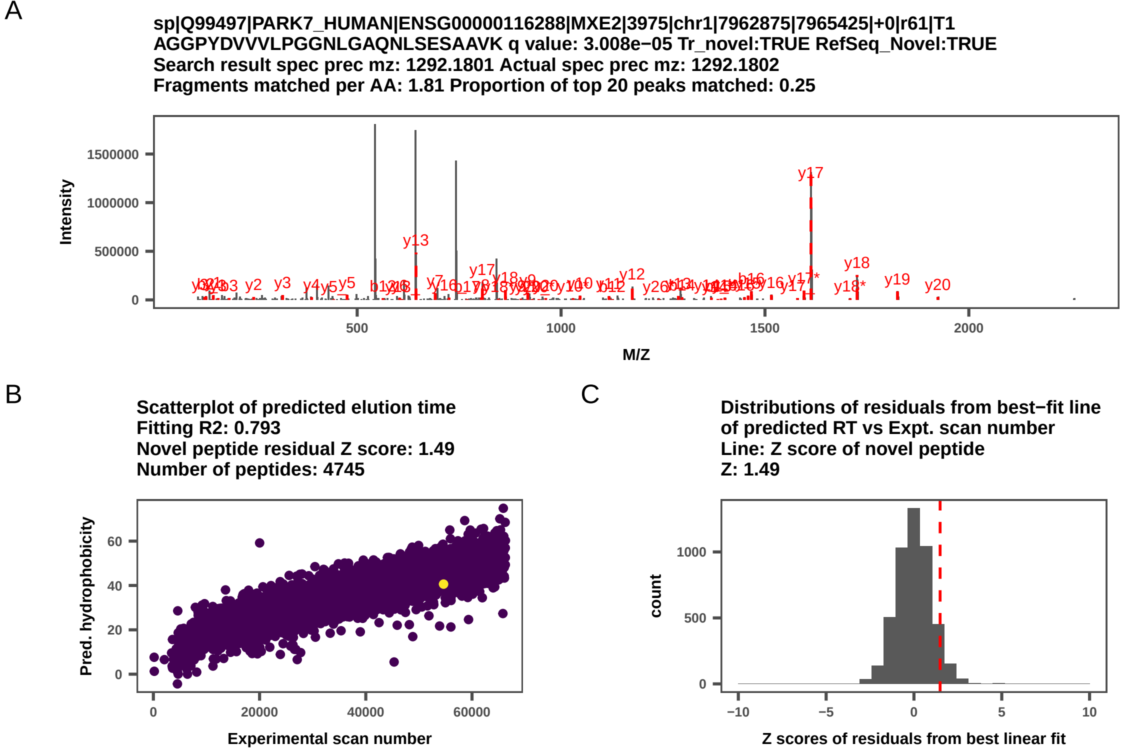
A
sp|Q99497|PARK7_HUMAN|ENSG00000116288|MXE2|3975|chr1|7962875|7965425|+0|r61|T1
AGGPYDVVVLPGGNLGAQNLSESAAVK q value: 3.008e−05 Tr_novel:TRUE RefSeq_Novel:TRUE
Search result spec prec mz: 1292.1801 Actual spec prec mz: 1292.1802
Fragments matched per AA: 1.81 Proportion of top 20 peaks matched: 0.25
Intensity
1500000
1000000
500000
0
y17
y13
y18
y19
y20
y2
y3
y5
y12
y18*
500
1000
1500
2000
M/Z
B
Scatterplot of predicted elution time
Fitting R2: 0.793
Novel peptide residual Z score: 1.49
Number of peptides: 4745
Pred. hydrophobicity
60
40
20
0
20000
40000
60000
Experimental scan number
C
Distributions of residuals from best−fit line
of predicted RT vs Expt. scan number
Line: Z score of novel peptide
Z: 1.49
count
1000
500
−10
−5
5
10
Z scores of residuals from best linear fit
